# Ecology and evolution of competitive trait variation in natural phytoplankton communities under selection

**DOI:** 10.1111/ele.14103

**Published:** 2022-09-27

**Authors:** Irene Gallego, Anita Narwani

**Affiliations:** ^1^ Department of Aquatic Ecology Swiss Federal Institute of Aquatic Science and Technology (EAWAG) Dübendorf Switzerland

**Keywords:** competition, evolutionary trait change, light, nitrogen, phosphorous, phytoplankton, R*, resource, Resource Competition Theory (RCT), traits

## Abstract

Competition for limited resources is a major force in structuring ecological communities. Species minimum resource requirements (R*s) can predict competitive outcomes and evolve under selection in simple communities under controlled conditions. However, whether R*s predict competitive outcomes or demonstrate adaptive evolution in naturally complex communities is unknown. We subjected natural phytoplankton communities to three types of resource limitation (nitrogen, phosphorus, light) in outdoor mesocosms over 10 weeks. We examined the community composition weekly and isolated 21 phytoplankton strains from seven species to quantify responses to the selection of R* for these resources. We investigated the evolutionary change in R*s in the dominant species, *Desmodesmus armatus*. R*s were good predictors of species changes in relative abundance, though this was largely driven by the success of *D. armatus* across several treatments. This species also demonstrated an evolutionary change in R*s under resource limitation, supporting the potential for adaptive trait change to modify competitive outcomes in natural communities.

## Graphical abstract

Natural phytoplankton communities were subjected to three types of resource limitation (nitrogen, phosphorus, light) in outdoor mesocosms and 21 strains were isolated to quantify responses to selection of R* for these resources in the lab. R*s of phytoplankton can be good predictors of responses to resource limitation in natural communities, though what determines success among relatively equal strong competitors with low resource requirements remains unknown. The dominant species demonstrated evolutionary change in R*s under resource limitation, supporting the potential for adaptive trait change to modify competitive outcomes in natural communities.

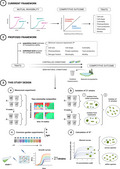

## INTRODUCTION

Resource availability fundamentally determines whether individuals of a species can meet their metabolic needs, grow and reproduce. If a competing species reproduces with less of a limiting resource, a species may be driven to extinction. As a result, resource limitation and competition for resources are amongst the most important drivers of community structure. Our understanding of competitive interactions remains largely in the realm of phenomenological description, and species' traits are most often invoked to explain the outcomes of competition rather than to make predictions (Adler et al., 2013). This may partly be attributed to the way in which outcomes of competition and its mechanisms are estimated. Best practices in estimating stable coexistence rely on the mutual invasibility criterion (Chesson, 2000; Grainger et al., 2019, Figure 1, part 1). The ability of species to mutually invade a community at equilibrium from which they are absent ensures stable coexistence, and the invasion rates can be partitioned to estimate the relative contributions of species niche and fitness differences to the competitive outcome (Gallego et al., 2019; Narwani et al., 2013). A subset of these investigations aims to identify traits associated with these outcomes and mechanisms (Gallego et al., 2019; Kraft et al., 2015; Kunstler et al., 2016; Spaak & De Laender, 2021). Whilst mutual invasibility is a general coexistence test encompassing all types of interactions (resource competition, allelopathy, apparent competition, etc.), the invasion rates measured for species in any particular community will not predict those of the same species in any other community configuration, nor even necessarily in the same community in a different environment *e.g.* under a different resource limitation. This approach must therefore by definition, be *post hoc*, because competition must happen before we can infer which mechanisms of coexistence—and ultimately traits—best explain the community assembly (Kraft et al., 2015; Gallego et al., 2019; Pérez‐Ramos et al., 2019, Figure 1, part 1).

**FIGURE 1:**
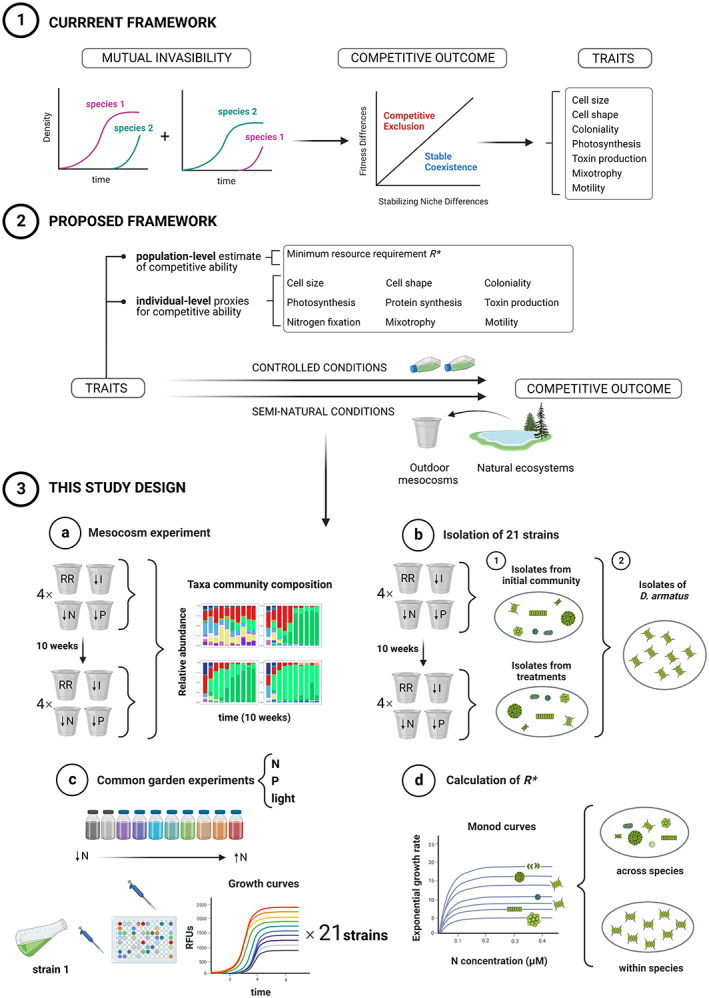
An overview of the most common current conceptual and methodological framework used to explain competition and coexistence (part 1), and an alternative framework that puts renewed emphasis on prediction (part 2). We present the workflow of this study based on the R* framework (part 3), which we propose because it has the potential to provide *a priori* predictions of the competitive community assembly under semi‐natural and natural conditions in the future. Traditionally, studies aiming to understand competitive interactions have been conducted as in scenario 1. Species' traits are often invoked to explain competitive outcomes rather than to make predictions, and the ability of species to mutually invade a community at equilibrium is interpreted as the difference between two mechanisms (*i.e.* stabilising niche differences and relative fitness differences) (Chesson, 2000). Traits are then quantified and tested as proxies for niche and fitness differences, and ultimately, as proxies for competitive outcomes. Examples of studies include Alexandrou et al., 2015; Funk & Wolf, 2016; Gallego et al., 2019; Godoy et al., 2014; Kraft et al., 2015; Narwani et al., 2013; Pérez‐Ramos et al., 2019 and Spaak & De Laender, 2021. Because this approach cannot be used to make predictions about new competition scenarios, we advocate for trait‐based approaches able to predict coexistence *a priori* (scenario 2). Amongst the most relevant phytoplankton traits mentioned, (Litchman & Klausmeier, 2008), we focus on the minimum resource requirement or R* (Tilman, 1982), as it is the only trait that directly quantifies the competitive ability of a species (although it must be measured at the population level). Laboratory controlled experiments have proven to accurately predict the outcome of competition in relatively simple communities (Ducobu et al., 1998; Grover, 1991a; Passarge et al., 2006; Sommer, 1986; Tilman, 1977). However, studies aiming to predict community assembly in naturally complex ecosystems and whether competitive ability traits evolve under resource limitations are lacking. Our study aims to quantify species' competitive traits (laboratory‐measured R*) may predict the outcome of competition of natural and diverse communities grown under semi‐natural conditions and variable climatic variations, providing greater prediction in studies of competition. The experimental design (3) includes four different steps: (a) Mesocosm experiment with four resource‐limitation treatments: light‐limitation (‘I’), nitrogen‐limitation (‘N’), phosphorus‐limitation (‘P’), and balanced resource‐supply (Redfield ratio, ‘RR’) over 10 weeks. Phytoplankton community composition was quantified weekly. (b) Isolation of a total of 21 strains (seven species) from the initial community and all four treatments at the end of the experiment. This included isolation of *D. armatus* strains from the initial community and all four treatments after selection. (c) Common garden experiments to determine the minimum resource requirement (R*) for each resource. Each strain was grown in 10 different levels of a resource to measure its growth curves. Relative Fluorescence Units (RFUs) were used as a proxy for algal biomass. (d) Calculation of R* for strain and resource, using Monod curves. We tested whether variation in R* values across species predicts success in competition for limiting resources. We compared R* values within the species *D. armatus*, to quantify evolutionary changes in competitive traits.

Tilman's Resource Competition Theory (RCT) proposes that species' minimum resource requirements (R*s) measured in isolation, can predict *a priori* the outcome of competition amongst species for a limiting resource (R) (Tilman, 1982, Figure 1, part 2). The species with the lowest R* for a given limiting resource is predicted to be the best competitor for that resource and should become the community dominant, or outcompete other species when that resource is limiting. In a meta‐analysis, ~75% of tests supported the predictions of Tilman's RCT (Miller et al., 2005), and of 25 studies focused on phytoplankton, only two did not provide strong support for RCT (Wilson et al., 2007). Much less is known about the usefulness of this trait‐based framework in predicting competitive outcomes in natural communities under variable environmental conditions, though it should be valid—but see Edwards et al., 2013a, 2013b and Edwards, 2016) (Figure 1, part 2). Predictions from RCT can also be converted to mutual invasibility outcomes according to Modern Coexistence Theory (Letten et al., 2017).

Since a population's R* has consequences for its competitive ability and ecological fitness in a community, we can expect this trait to evolve in response to selection by resource limitation (Chase & Leibold, 2003). R* can be used to evaluate the outcome of competition between genotypes within populations *via* lineage sorting (Chase & Leibold, 2003), as competition between asexual lineages is not distinguished from competition between species. Whilst it is well known that phytoplankton traits can evolve rapidly enough to influence consumer‐resource dynamics (Jones et al., 2009; Yoshida et al., 2003, 2007), and respond to abiotic perturbations (Thibodeau et al., 2015), less is known about how competitive traits evolve in response to competition under resource limitation. In one prior study, we showed that R*s of a single species can evolve under selection by resource limitation (Bernhardt et al., 2020). It remains unknown whether such trait evolution occurs in natural communities.

Trade‐offs in R*s for different resources amongst species (or populations) are required to explain the stable coexistence of multiple species in a single environment, and species turnover across environments with different limiting resources (Tilman, 1982). This assumes that species that are good competitors for one resource (*e.g.* nitrogen) are worse competitors for another resource (*e.g.* phosphorus) because investment in one physiological function comes at an energetic or material cost to another (Sterner & Elser, 2002). Whilst trade‐offs are a prerequisite for the competitive coexistence of multiple species, the existence of such trade‐offs in R* has rarely been documented, particularly amongst populations selected under different resource‐limitation regimes —but see Edwards et al., (2011).

The goal of this study was to determine whether R*s measured under controlled laboratory conditions, can explain species' performance and competitive outcomes under single‐resource limitations, under more natural conditions (Figure 1, part 3). Specifically, we assess whether species with lower R*s are better competitors and increase in relative abundance over time in semi‐natural outdoor mesocosms (open to dispersal), where we impose selection by a single limiting resource (light, nitrogen or phosphorus), but where we allow resource concentrations to fluctuate and the climate to vary. We also investigate whether R*s can evolve in such natural communities in response to selection by single resource limitation. Specifically, we test whether R*s of single species measured in the lab decline over time in resource‐limited conditions, and whether they become lower than those observed in a non‐limiting control. Finally, we examine the evidence for the existence of trade‐offs amongst R*s or *μ*_max_ amongst species and populations selected under three unique limiting resource environments.

We performed an outdoor mesocosm experiment with natural phytoplankton communities in which we imposed four treatments over 10 weeks: (1) balanced resource supply (Redfield ratio), (2) light‐ (3) nitrogen (N)‐ and (4) phosphorus (P)‐limitation (Figure 1, part 3a). We then tested whether these resource limitation treatments resulted in improved performance of taxa with lower R*s for the limiting resources. We isolated 21 phytoplankton strains comprising seven different species from the initial community and the four treatments at the end of the experiment (Figure 1, part 3b). We then quantified these strains' R*s for light (I*), nitrogen (N*) and phosphorus (P*) using common garden experiments in the lab (Figure 1, part 3c). For one species, *Desmodesmus armatus*, we were able to isolate strains from the initial time point and all treatments at the end of the experiment, enabling an investigation of trait evolution for ~70 generations (Figure 1, part 3d). We predicted that lab‐measured R*s for individual resources would be good predictors of species' performance in competition for the same limiting resource in semi‐natural mesocosms. More specifically:

1. Species with lower R*s for any single limiting resource will increase more in relative abundance over time and become dominant.
2. Under a balanced resource supply, species with higher maximum specific growth rates (*μ*_max_) will increase more in relative abundance over time.
3. Adaptive evolution in response to resource limitation will cause species' R*s for the limiting resource to decline.
4. There will be negative correlations (trade‐offs) amongst R*s for different resources, as well as trade‐offs between R*s and *μ*_max_.

## METHODS

### Mesocosm experiment and treatments

We established an outdoor mesocosm experiment with 16 cylindrical polyethylene tubs (volume = 110 L, depth = 0.7 m) at our experimental facilities at Eawag (Dübendorf, Switzerland). The mesocosms were filled with surface water from Lake Greifen (47.3478° N, 8.6793° E, NO_3_‐N = 1.6 mg/L, PO_4_^3−^P = 1.7 μg/L at the beginning of the experiment), after pre‐filtration of the water through a net (mesh size 110 μm) to remove large zooplankton. Our experimental resource manipulations aimed to create and maintain a single‐limiting resource (or balanced resource supply as a control) in each mesocosm by manipulating the ratios of the total nitrogen and phosphorus in the medium, and the availability of light. The mesocosms were randomly assigned an experimental treatment: control with balanced nutrient supply (Redfield ratio = 16:1 molar ratio), nitrogen limitation (‘N‐lim’, N:P = 8:1), phosphorus limitation (‘P‐lim’, N:P = 32:1) and light limitation (‘L‐lim’, N:P = 16:1 but reducing the upcoming irradiance using neutral density filters). We replicated each treatment four times.

Lake Greifen has naturally high nitrate levels, so we first diluted the lake water with DI water in 1:1 ratio to reduce the total nitrogen concentrations. We added stock solutions of phosphate (K_2_HPO_4_) or nitrate (NaNO_3_), to achieve targeted N:P molar ratios. We immersed a water pump in each mesocosm to homogenise and mix the water. We measured the irradiance and water temperatures in each mesocosm weekly. We took 10 L water samples weekly to measure nutrients, chlorophyll *a* and species counts, and then replace the water that was removed (details below). The experiment lasted 10 weeks (July 7 – September 18, 2019; Figure 1, part 3a). Chlorophyll *a* and phytoplankton total biovolume reached steady‐state in week 7 (see SI1A Figure S1 and SI2 Figure S1).

To ensure that the treatments (*i.e.* total resource ratios) were maintained over time, we measured the total and dissolved concentrations of N and P and irradiance. We then added nutrients (dissolved in 10 L of DI water) weekly to achieve total concentrations of 160 μM of N (80 μM under N‐limitation) and 10 μM of P (5 μM under P‐limitation) and the desired molar ratios. We controlled the total resource concentrations and resource ratios to maintain the identity of the limiting resource over time, and to coarsely mimic the selective resource conditions of a chemostat. There are noteworthy differences between chemostats and outdoor mesocosms in terms of experimental control. In chemostats, the nutrient inflow and outflow rates are continuous and constant. Consequently, total resource concentrations and ratios are also constant. In open mesocosms, rain, evaporation and organismal immigration, make nutrient inflow and outflow rates uncontrolled and variable. Hence, we aimed to maintain selection by a single limiting resource in the mesocosms by maintaining constant total resource concentrations (dissolved + particulate) and resource ratios on average over time using periodic adjustments based on measured levels (*e.g.* Levine & Schindler, 1999, Hall et al., 2004, 2005; SI1A Figure S2). We performed a rm‐ANOVA to ensure that N:P ratios and irradiance were maintained over time and differed between the treatments (SI1A Figure S1).

### Isolation of strains

Algal strains were isolated from the mesocosms at two different time points: at the beginning of the experiment, before the treatments were applied (initial community), and at the end of the experiment, with populations that had grown under the four different treatments for 10 weeks (L‐lim, N‐lim, P‐lim, Redfield) (Figure 1, part 3b). The dominant species at each time point and in each treatment were isolated by combining two approaches: (1) Cell‐sorting based on size and fluorescence using flow cytometry (aBD Facs Aria III BSL1 sorter, Cytometry Core Facility, ETH Zürich). (2) Serial dilution, filtration and micropipetting under a microscope, to isolate large taxa (>40 μm) (Andersen, 2005). We isolated 21 strains (started from unique isolates) from seven different species —10 strains from the initial community and 11 from different treatments at the final time point—. This included eight strains of the same species, *Desmodesmus armatus*. For this species, we estimated R*s at the start and at the end of the mesocosm experiment (from all treatments) to investigate evolutionary trait change. We maintained batch cultures of all isolated strains in COMBO medium (Kilham et al., 1998) at low temperature and irradiance (14°C, 20 μmol·m^−2^·s^−1^) to limit growth until we started the R* experiments.

### Laboratory experiments and determination of R*, cell size and stoichiometry

We conducted three independent common garden experiments to determine the R*s for each strain for each resource (*e.g.* light, nitrogen and phosphorus). We calculated I* as the light compensation point, *I*_*c*_, sensu Huisman and Weissing (1994). We measured the growth rates of each strain at 10 different resource levels for each resource over 2 weeks (Figure 1, part 3c; Figure 3, SI1C). Additionally, we measured cell size and biomass stoichiometric ratios (C:N and C:P) for each strain under balanced resource supply and under limitation by each resource. Strains were acclimated to resource limitation treatments for 72 h (*ca*. 3 generations). Detailed methods of the common garden experiments are available in SI1B.

We modelled population growth using the ‘growthTools’ package in R (Kremer, 2020). We estimated *per capita* population‐level growth rates for each replicate of each resource treatment separately, by fitting multiple possible models to the fluorescence time series. The model alternatives varied from a simple exponential growth model by including a lag or a saturated‐growth phase, or both. We selected the best‐fitting model based on Akaike's Information Criterion, corrected for small sample sizes (AICc). We then fitted the Monod model to the individual growth rates as a function of resource concentration, R:

(1) $$\mu \left(R\right)=\mu_{\max}\;\left(R/\left[K_{s}+R\right]\right)$$

where *μ* is the population growth rate, *μ*_max_ the maximum population growth rate (both are *per capita*), and *K*_*s*_ the half‐saturation constant. We estimated the minimum resource requirement, R*, from the parameters in the previous equation as follows:

(2) $$R^{*}=\left(m\;K_{s}\right)/\left(\mu_{\max}-m\right)$$

where *m* is the density‐independent mortality rate, set at 0.013 day^−1^ to reflect the mortality caused by dilution (a weekly discrete dilution of 10 L) in our mesocosm experiment (see Figure 3 and SI1C).

### Statistical analyses

To test whether phytoplankton community composition from the mesocosms differed between treatments over time, we performed an ANOSIM with 999 permutations. We calculated Indicator Value species (IndVal) using 999 permutations to test for associations between species and treatment(s) (Legendre & Legendre, 2012). To determine whether phytoplankton structure changed over time under different treatments, we performed a Community Trajectory Analysis (CTA) with Hellinger‐transformed species abundances. We used Non‐metric Multidimensional Scaling (NMDS) to observe how phytoplankton community ordinations (based on species abundances) varied over time (week 1, week 5, week 10). We used the ‘vegan’ R package for the above‐mentioned tests.

We tested prediction 1 (*i.e.* species with lower R*s have greater increases in relative abundance over time and become dominant) to determine how well species R*s corresponded with mesocosm performance. To do so, we fitted a linear mixed effect model, where we included the identity of the resource for which R* was estimated (light, nitrogen, phosphorus) as a random effect (‘nlme’ R package). R*s for the three resources were z‐score transformed. When multiple strains of the same species were isolated, we calculated the mean of their R*s for each resource. Residuals were examined to check for normality and homoscedasticity. To test prediction 2 (*i.e.* species with higher *μ*_max_ values show greater increases in relative abundance over time under balanced supply), we tested for a positive association between species' changes in relative abundance over time under balanced supply (Redfield ratio) and their maximum growth rate (*i.e.* maximum *μ*_max_ across all experiments) using Spearman rank correlations. To test prediction 3, (adaptive evolution in response to resource limitation causes R*s to decline over time in *D. armatus*), we performed ANOVAs using mesocosm selection treatment as a predictor and R* as a response variable for each resource. We used Tukey post hoc tests to compare the R* in the selection treatment at the final time point to the R* at the initial time point (before evolution) and to the final time point under balanced resource supply (after evolution, in the absence of limitation by a single resource). Finally, to test prediction 4 that trade‐offs exist between R*s (I*, N*, P*), cell size and *μ*_max_ *max*, we investigated pairwise correlations between those traits (log_10_‐transformed) and partial correlations with one, two or three covariates.

## RESULTS

### Change in community composition over time

**FIGURE 2:**
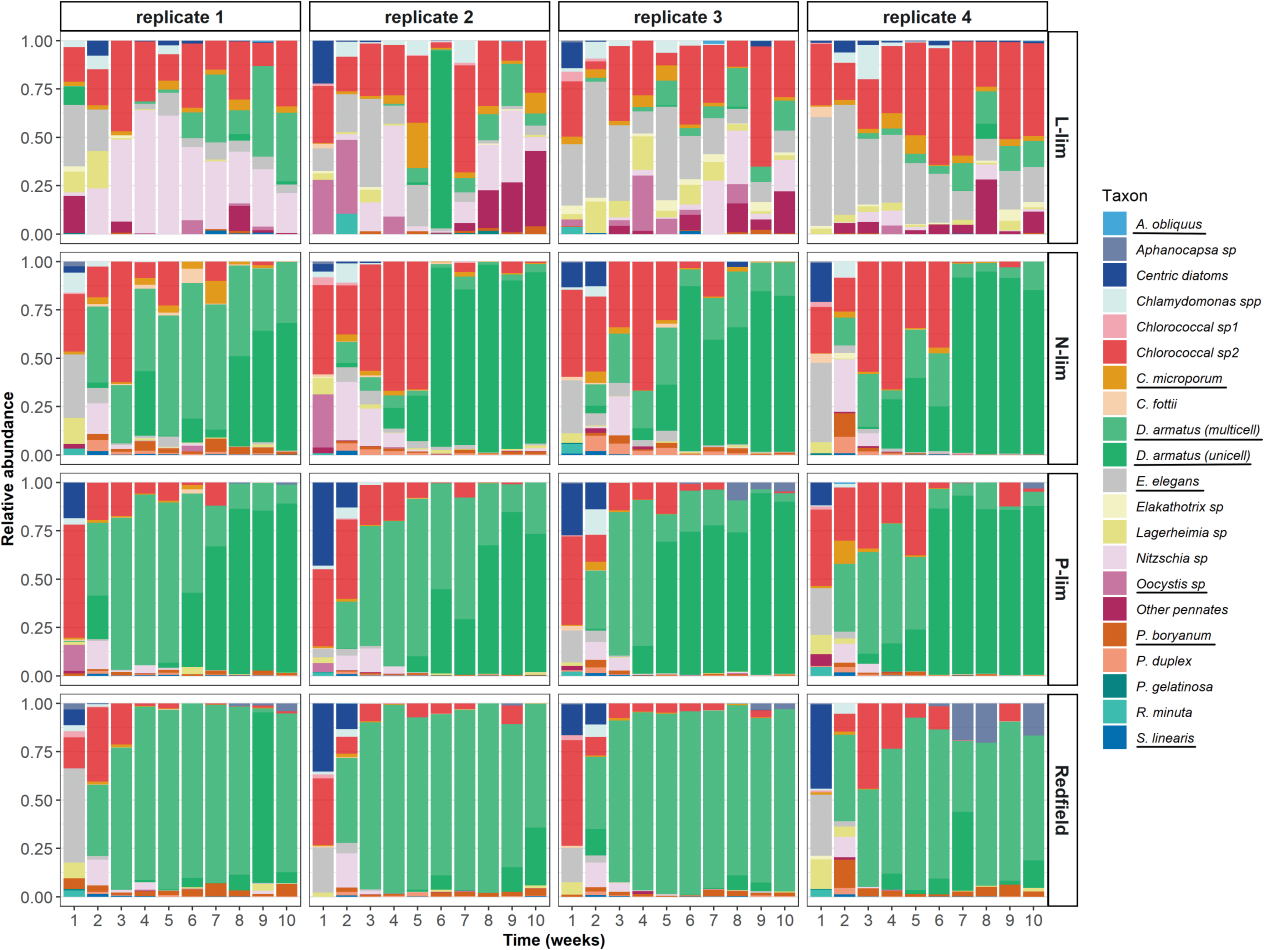
Phytoplankton community composition over 10 weeks. The X axis represents time in weeks, whilst Y axis represents taxa relative abundance per mL. Species that were isolated for laboratory experiments are underlined. The dominant species, *Desmodesmus armatus*, was also accounted for by its two different morphotypes in green colours, *i.e.* *D. armatus* (multicellular) and *D. armatus* (unicellular).

Phytoplankton communities all started out with similar taxonomic richness, evenness and species identity (Figure 2, SI2 Figures S1 and S2) and showed significant changes in community structure over time (ANOSIM *R* = 0.2899, *p*‐value = 0.001; Figure 2, SI2 Figures S3 and S4). By the end of the experiment, phytoplankton community structure differed significantly between treatments (ANOSIM *R* = 0.3981, *p*‐value = 0.001; Figure 2; SI2 Figures S3 and S4). Communities under light‐limitation showed similar evenness over time and were co‐dominated by diatoms and chlorococcales (Figure 2, SI2 Figure 1). Phytoplankton communities in the N‐lim, P‐lim and Redfield treatments shifted from high species evenness to the dominance of *Desmodesmus armatus* (Figure 2, SI2 Figures S1 and S2a). N‐lim and P‐lim treatments were dominated by a unicellular morphotype, whilst the balanced resource supply and L‐lim were both dominated by *D. armatus* (multicellular morphotype) at the end of the experiment (Figure 2, SI2 Figures S1 and S3). Unicellular *D. armatus* was the indicator of N‐lim and P‐lim treatments at the final time point of the experiment (week 10, IndVal *p*‐value = 0.001).

**FIGURE 3:**
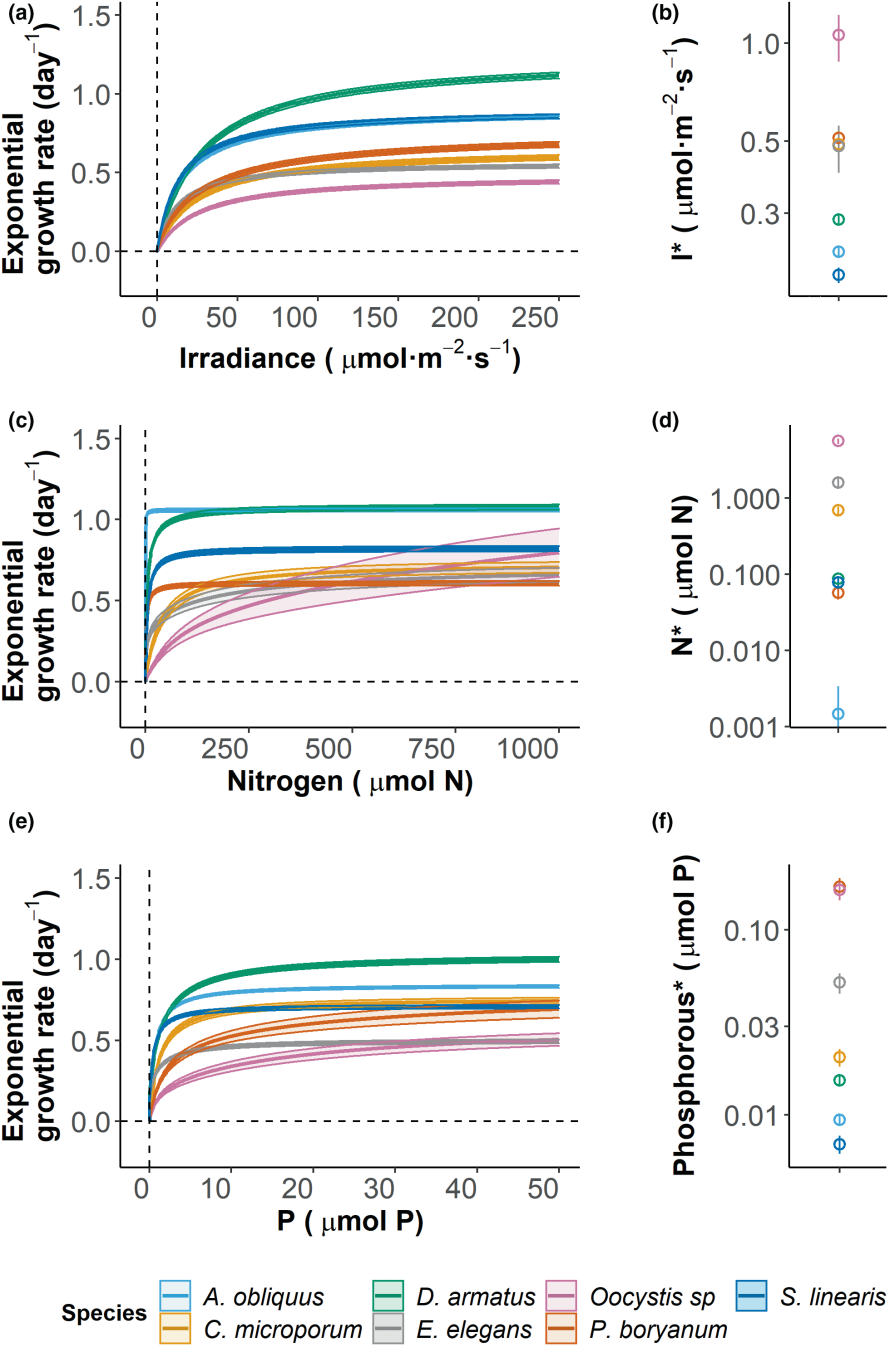
Monod curves (left panels) for each species and each resource estimated by fitting exponential growth rates to resource availability, and the resulting estimates of minimum resource requirement ‐or R* values‐ for each resource (right panels). The resources are light (or irradiance, panels a & b), nitrogen (c & d), and phosphorous (e, & f). Central curves or plotted points represent average estimates for each species. Error bars or ribbons represent ± SE.

**FIGURE 4:**
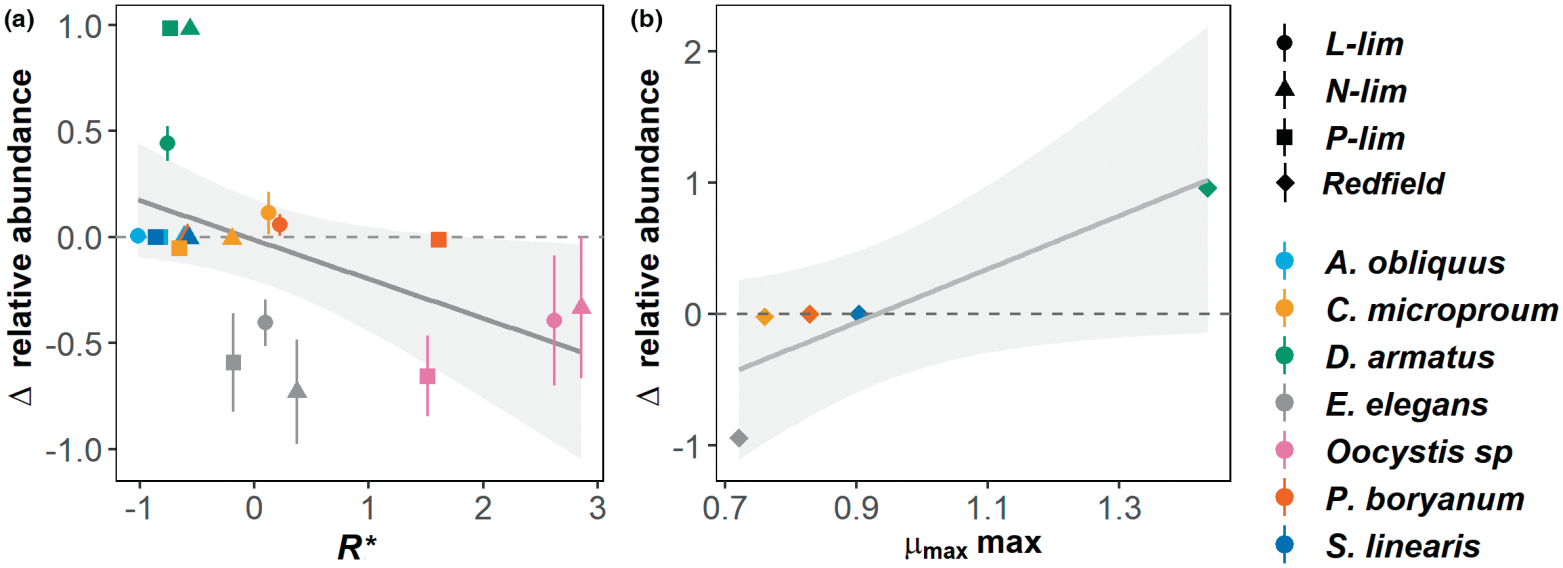
Changes in species relative abundances over time (*t* = 10 weeks) are negatively associated with species' z‐score mean standardized trait values R* under different limiting resources (y = −0.012–0.185x, *R* ^2^ = 0.216, *p* < 0.05, panel a). and were positively associated with the species maximum growth rate (*μ*_max_max), under balanced supply (Spearman ⍴ = 0.943, *p*‐value <0.05, panel b). Error bars represent differences in relative abundances per replicate (*n* = 4) for each treatment. Distinct shapes represent the different treatments, whilst different colours represent species. ∆ relative abundance represents the change in species relative abundance between week one and week ten. L‐lim = light limitation, N‐lim = nitrogen limitation, P‐lim = phosphorus limitation, Redfield = Redfield ratio.

**FIGURE 5:**
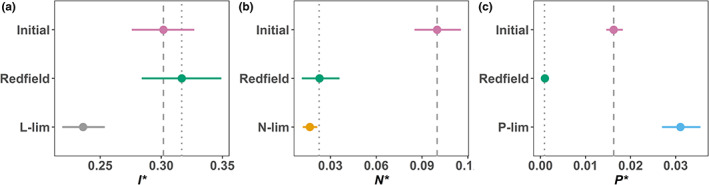
Competitive ability traits I* and N* of *Desmodesmus armatus* show evolutionary variation and decrease their values under light‐ and nitrogen‐limited environments respectively, whilst P* increases under phosphorus limitation. The X axis shows the values of the traits measured (I*, N*, P* in panels a–c respectively). The Y axis represents the different treatments and conditions, where “Initial” represents the initial phytoplankton community (grey dash line), “Redfield” is balanced resource supply (grey dotted line), and “L‐lim”, “N‐lim”, “P‐lim” represent the resource limitation treatments (light, nitrogen and phosphorus, respectively). Points represent the mean R* values for each treatment, with 95% CI.

**FIGURE 6:**
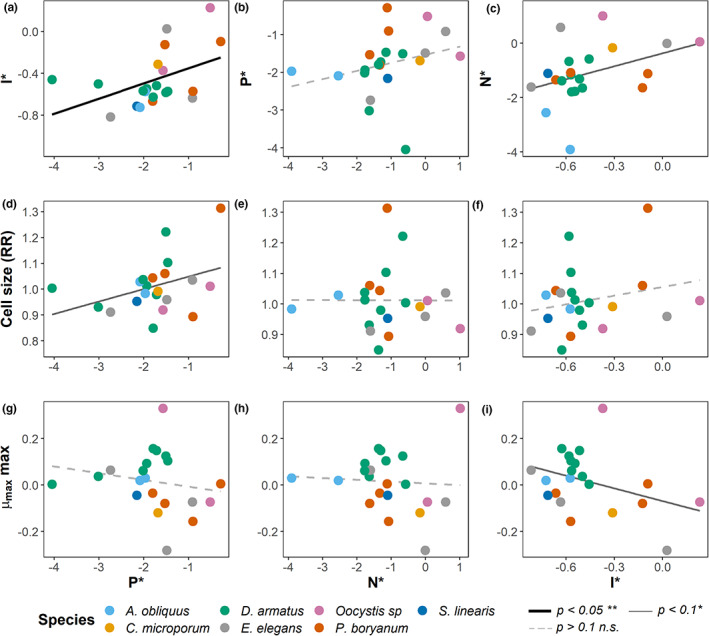
Bivariate scatter plots depicting positive associations between the competitive ability traits P* and I* (panel a), and between N* and I* (c). The competitive ability for phosphorous P* was also positively correlated with the mean cell size for each species under balanced resource supply (d). I* and species maximum growth rate (*μ*_max_ *max*) are negatively correlated (i). Each point represents a strain isolated from the mesocosm experiment, coloured by their taxonomic identification. Variables were log_10_ transformed. Significance: **p < 0.05, *p < 0.1, *n.s. p* > 0.1. (Complete model results in Supplementary Information SI2 Table S1).

### Support for predictions 1 and 2: Species with lower R*s increase their relative abundances; species with higher *μ*_max_ under balanced supply dominate

Increases in species' R*s predict reduced competitive success, as changes in relative abundance over time were negatively related to species R*s (y = −0.012^ns^ ‐ 0.185x**, *R* ^2^ = 0.216, Figure 4a). This relationship was largely driven by two taxa: *D. armatus* and *Oocystis* sp. *D. armatus* had low R*s for all three resources (Figure 4a, SI2 Figure S5) and had large positive increases in relative abundance, particularly under nitrogen and phosphorus limitation. *Oocystis* sp. had the largest R*s on average and displayed relatively large, negative changes in relative abundance. Species maximum growth rates (*μ*_max_max) were positively correlated with the changes in relative abundance under balanced supply, as predicted (Spearman ⍴ = 0.943, *p* <0.05, Figure 4b).

### Support for prediction 3: Adaptive evolution under resource limitation causes a species' R* to decline for the limiting resource

We investigated evolutionary trait change in a single species isolated from all treatments, *D. armatus*. Populations displayed significant trait variation between time points and conditions of selection for I*, N* and P* (*F* = 5.025, *p* < 0.05; *F* = 84.56, *p* < 0.001 and *F* = 64.63, *p* < 0.001, respectively). Changes in R* in *D. armatus* were consistent with our expectations of adaptive evolution of resource competitive abilities under selection by light and nitrogen limitation, as these treatments showed reductions in I* and N*, respectively, over time (Tukey‐HSD_L‐lim‐Initial_ *p* = 0.053, Tukey‐HSD_N‐lim‐Initial_ *p* < 0.001; Figure 5a,b). The I* of low‐light selected *D. armatus* was also lower than for the population selected under balanced resource supply (Tukey‐HSD_L‐lim‐Redfield_ *p* < 0.05; Figure 5a). N* was significantly lower under N‐lim than under initial conditions (Tukey‐HSD_N‐lim‐Initial_ *p* < 0.001; Figure 5b), although no significant differences were found between strains growing with limited nitrogen and Redfield ratio (Tukey‐HSD_N‐lim‐Redfield_ *p* > 0.1; Figure 5b). Unexpectedly, the *D. armatus* strain isolated under P‐limitation had a higher P* than the strain isolated under the Redfield ratio (Tukey‐HSD_P‐lim‐all_ *p* < 0.001; Figure 5c) or from the initial community (Figure 5c). *D. armatus* grown under P‐limitation had lower C:P ratios than the strain isolated from the Redfield ratio condition (20% reduction when they were grown under Redfield ratio, and 70.5% reduction when they were grown under P‐lim; SI2 Figure S6). Thus, adaptation to low P does not result in a lower P*, but rather in larger cell size and lower C:P ratio.

### Support for prediction 4: Trade‐offs amongst traits

Correlation and partial correlation analyses of traits of all isolated strains from the mesocosm experiment indicate no support for trade‐offs in competitive abilities for different essential limiting resources (Figure 6, SI2 Tables S1 and S2). Instead, a positive relationship between P* and I* (y = −0.2045 + 0.1456x, *p* < 0.05, Figure 6a) and marginally, between N* and I* (y = −0.3681+ 1.5904x, *p* < 0.1, Figure 6c) were observed. P* was also positively but marginally correlated with cell size (y = 1.0971 + 0.0482x, *p* < 0.1, Figure 6d). I* and species maximum growth rate across all experiments (*μ*_max_ *max*) were negatively correlated, suggesting that the best competitors for light are also the fastest growing species under unlimited light (y = −0.0689 ‐ 0.1822, *p* < 0.1, Figure 6i; SI2 Table S1).

## DISCUSSION

Trait‐based approaches to predict the outcome of competition in natural communities are currently lacking. We propose Tilman's R* framework as a useful approach to move beyond phenomenological *post hoc* description, and towards making predictions of competitive community assembly, particularly for outdoor natural communities of phytoplankton. Our results are broadly consistent with the idea that species with low laboratory‐measured R*s for any given resource will increase in relative abundance in (semi‐)natural conditions when that resource is limiting. However, this predictive relationship was largely driven by the success of one highly competitive species, *D. armatus* and the demise of one poor competitor, *Oocystis* sp. Some species with low R*s did not increase in relative abundance, indicating that resource competitive ability may not be the only determinant of success in the mesocosms. We did not find that a different species dominated in each resource condition. Instead, one species, *Desmodesmus armatus*, was successful in dominating communities under three scenarios of resource availability, in line with the lack of support we found for the common assumption that trade‐offs exist amongst taxa. Whilst we were only able to evaluate trait evolution in one species, and therefore cannot compare it to the adaptive potential of other species, it is possible that this particular species was successful in multiple scenarios due to initially high‐standing genetic variation and ability to respond to selection (Lürling, 2003).

Our study shows that resource limitation impacts both phytoplankton community composition and diversity and that the identity of the limiting resource matters. Species evenness dropped drastically over time under nitrogen‐ and phosphorus‐limited conditions, in line with the competitive exclusion principle. However, this decline in evenness was not observed when the light was limited; in fact, evenness was higher in this treatment at the end of the experiment than at the start. This may be because light is a resource that can be partitioned, with taxa specialising on different wavelengths (Stomp et al., 2004, 2007), enabling coexistence. Interestingly, community compositions did not differ when nitrogen or phosphorus were limited. This outcome occurs because the same species, *D. armatus*, was successful in both treatments. It also results from the lack of a trade‐off in R* for these two resources.

More generally, we did not observe trade‐offs in competitive abilities for different resources, but instead found a positive association between competitive abilities for light (I*) and phosphorus (P*). This finding is in broad agreement with previous studies in which selection under different types of essential resource limitations led to convergent evolution in core metabolic efficiency (Tamminen et al., 2018), and positive correlations in R*s (Bernhardt et al., 2020). Phosphorus requirements were also positively associated with cell size, which may be explained by allometric scaling: smaller phytoplankton will have higher surface‐to‐volume ratios allowing greater rates of resource uptake per unit of biomass (Irigoien et al., 2004; Raven, 1998) —but see Acevedo‐Trejos et al. (2015). The observed lack of trade‐offs in our study may be surprising, given that the evolution of resource‐use partitioning traits and trade‐offs explains the diversity in some well‐known systems (Grant, 1999; Rainey & Travisano, 1998; Schluter, 1995; Seehausen, 2015). However, phytoplankton competes for a limited number of essential resources, which limits how much resource partitioning is possible, and therefore, the diversity of species that can coexist *via* resource‐use partitioning (Fox & Vasseur, 2008). This means that competitive resource‐use partitioning for nitrogen and phosphorus is unlikely to explain the high diversity of phytoplankton observed in our initial community from Lake Greifen, whilst resource‐use partitioning for light may provide greater explanatory power (Stomp et al., 2004; Stomp et al., 2007).

When we studied within‐species competitive traits, we found that *D. armatus* was able to lower its I* and N* significantly when selected under low light and nitrogen, respectively. This ability of *D. armatus*' competitive traits to evolve rapidly in response to resource availability may explain why this species became dominant under multiple limiting conditions, though we cannot confirm this because we were unable to compare this to any other taxon in the community. *Desmodesmus* has the ability to recombine sexually (Lürling, 2003), which might contribute to genetic variation and enable rapid adaptation under stress (Thibodeau et al., 2015). In our experiment, *D. armatus* shows a low R* under multiple resource‐limiting conditions and a fast growth rate when resources are abundant. Together, this led to near competitive exclusion within the communities in three of four resource environments. *D. armatus* has a generation time of ~24 h, and trait evolution occurred within 70 generations of low‐resource selection. Phenotypic evolution in natural phytoplankton species (including *Desmodesmus*) has been reported at similar timescales in response to acidification (Thibodeau et al., 2015). *Chlamydomonas reinhardtii* also reduced its resource requirements in response to resource limitation in lab experiments over ~285 generations in the absence of sexual recombination (Bernhardt et al., 2020).

Surprisingly, *D. armatus* increased its minimum requirement for phosphorus (P*) under phosphorus limitation but became dominant at the community level, counter to our hypothesis. The low‐phosphorus selected *D. armatus* strain also showed lower C:P ratios, both when later grown under phosphorus limitation and under balanced resource supply (SI2 Figure S5; see also Grover, 1989). This suggests that the low‐phosphorus treatment in the mesocosm experiment selects for a phosphorus‐storage phenotype rather than a fast‐growing phenotype. Cells may accumulate phosphorus under low supply and invest in cell growth rather than in cell division, in agreement with the positive P*‐cell size correlation across species. Phytoplankton is notoriously plastic in their cellular nutrient ratios, both at the species and community level (Guildford & Hecky, 2000; Hillebrand et al., 2013; Sterner et al., 1998). Inorganic phosphorus storage can happen in both high‐ and low‐phosphorus environments and may be associated with larger cells in various taxa (Lin et al., 2016). This is likely because phosphorus limitation arrest DNA replication and, therefore, cell division (Joint, 1995). However, increases in cell size should decrease phosphorus uptake rates per unit of cell volume (Edwards et al., 2011), and thus be disadvantageous under phosphorus‐limited conditions. A counter‐adaptation to increased cell size may be to increase polyphosphate storage capacity (Kornberg et al., 1999), which may increase the competitive ability (Grover, 1991b) but it would be observed as an increase in P* since cells grow larger, divide less and require more phosphorus after selection under low‐phosphorus. Such an adaptive shift in life history strategy may exemplify a limitation of R* estimates from Monod curves to predict competition amongst populations within a species. The use of the Droop formulation of resource‐dependent growth, which explicitly deals with nutrient storage (Droop, 1973), may be a solution in future studies.

Our study advances our understanding of trait‐based competitive community assembly in phytoplankton, but it has some limitations: (1) We isolated an unprecedented number of phytoplankton strains (21) from a natural community and estimated their R*s for three essential resources. Nevertheless, we were not successful in isolating all taxa present in the light treatment, *e.g.* pennate diatoms, and we would ideally have examined trait evolution in more than one species. It would also have been preferable to have multiple replicate strains of *D. armatus* from each treatment, *e.g.* one per replicate mesocosm. We reached the limit of our logistical capacity, as this work is laborious, and many species were difficult to culture. Automation and resulting greater replication may increase the chances of success in future efforts to culture wild populations. (2) The R* framework is focused on equilibrium conditions, *i.e.* constant resource supply. However, our mesocosms experienced ambient natural variation in temperature and light, as well as weekly amendments of resources. Despite deviations from the assumption of equilibrium and stable environmental conditions, the across‐species traits worked relatively well in explaining community assembly. (3) It could be argued that only a limited set of environmental conditions were investigated and that trade‐offs might have been discovered along other gradients of environmental variation, e.g. silicon or iron availability, grazing, temperature, pH or turbulent mortality. For example, a competition‐defence trade‐off might explain why the slow‐growing *Oocystis* sp is a poor competitor for all resources. This would need empirical evaluation, however, as *D. armatus* is also known to induce defence against Daphnia grazing (Lürling, 2003). Regarding our selection of nutrients, there is extensive evidence for nitrogen and phosphorus as important drivers of phytoplankton species composition, and therefore good justification to investigate these resources (Burson et al., 2019; Klausmeier et al., 2004). (4) It is possible that under balanced supply (Redfield ratio), multiple resources may become limiting, causing co‐limitation (Price & Morel, 1991) rather than selection for fast‐growing species, as we hypothesised. In our balanced supply treatment, nutrient levels reached limiting values (SI1A Figure S2), suggesting co‐limitation. Nevertheless, model comparisons showed that *μ*_*max*_ was the best predictor of changes in relative abundance over time (SI2 Table S2).

In this study, we found that R*s of phytoplankton can be good predictors of responses to resource limitation in natural communities, though what determines success amongst relatively equal strong competitors with low resource requirements remains unknown. Tilman's RCT may therefore present a way to move competition theory from *post hoc* phenomenological description towards prediction, though we acknowledge that measuring R*s is labour‐intensive and perhaps not feasible for longer‐lived, slow‐growing organisms (but see Dybzinski & Tilman, 2007). The predictive ability of R* in this study was largely driven by the success of one species across multiple limiting resource environments. This species also demonstrated significant evolution of competition traits. Overall, we suggest that quantifying species' competitive traits is an important step towards greater prediction in studies of competition, though it may only provide predictive ability across large gradients of variation in species‐level R*s. Furthermore, our study suggests that there is a high potential for rapid local adaptation of these traits that may impact the outcome of competitive community assembly, resulting in eco‐evolutionary dynamics of resource competition in phytoplankton. Such dynamics may ultimately limit the predictive capacity of R*s, without adequate predictions about the evolutionary trajectories of these traits. Finally, we recommend that when such traits are used for prediction, they be measured on populations isolated from the local environments in which predictions are to be made to account for the potentially important role of local trait adaptation.

## AUTHOR CONTRIBUTIONS

AN conceived the original idea. AN and IG designed the study. IG performed the mesocosms and laboratory experiments, analysed data, made figures and drafted the manuscript. AN contributed significantly to data interpretation and commented on manuscript drafts.

### PEER REVIEW

The peer review history for this article is available at https://publons.com/publon/10.1111/ele.14103.

## Supporting information


Data S1

Data S2

## Data Availability

The data supporting the results will be archived at Dryad as: Gallego, Irene; Narwani, Anita (2022), Ecology and evolution of competitive trait variation in natural phytoplankton communities under selection, Dryad, Dataset, https://doi.org/10.5061/dryad.dz08kps0x and at ERIC‐open (Eawag Research Data Institutional Collection).
