# Supplementary material for: Ecology and evolution of competitive trait variation in natural phytoplankton communities under selection
Source: Ecol Lett. 2022 Sep 27;25(11):2397–409. doi: 10.1111/ele.14103 (PMC9828480; doi:10.1111/ele.14103)

**Supplementary Information 1**

1. Effectiveness of the experimental treatments
2. Methodology of the laboratory experimental design
3. Monod curves for *I*, N** and *P** for the seven studied species

**A. Effectiveness of the experimental treatments**

The N:P ratios differed significantly between treatments, with the N:P ratio being the highest under P-limitation, lowest under N-limitation, and moderate in the Redfield and light limitation treatments (SI 1A Fig. 1a, b, adj-p value < 0.001). The N:P ratios did not differ significantly, except between the light limitation and Redfield ratio treatments (adj-p value >0.1). The N:P ratios did not differ significantly over time in any treatment (p value > 0.1 in all cases, SI 1A Fig. 1a, b). Irradiance levels were significantly lower in L-lim than in other treatments (adj-p value < 0.05). Irradiance levels did not vary significantly over time under light limitation (adj-p value > 0.1), and differences over time in other treatments were caused by the high irradiance levels occurring at week 1 (adj-p value< 0.05 in all cases; SI 1A Fig. 1c, d). Chlorophyll *a* levels also varied significantly over time in all treatments, with the exception of the L-lim treatment (adj-p value > 0.1), but post hoc tests show that there were no significant differences over time after week 5 and later weeks (adj-p value > 0.05, SI 1A Fig. 1e, f). This suggests that communities had reached steady state biomass in all treatments.


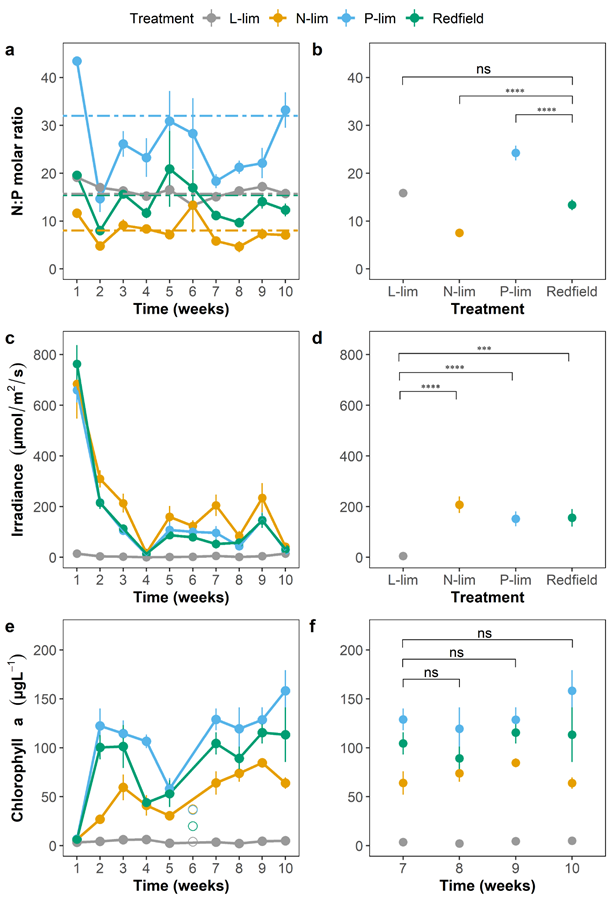


**SI 1A Figure 1.** Mean value of N:P molar ratios (panels a, b), irradiance measured in air and water (panels c,d), planktonic chlorophyll *a* (panels e,f) for each treatment used in our mesocosm experiment over 10 weeks. Dashed lines in panel a represent the targeted N:P ratio for each treatment (N:P = 32 in the phosphorus limitation treatment, N:P = 16 in light limitation and Redfield ratio treatments, N:P = 8 in nitrogen limitation treatment); whilst solid lines represent the mean N:P ratio over time for each treatment. Empty circles represent potential sampling errors. Error bars represent differences in each measured variable per replicate (n=4) for each treatment.

L-lim = light limitation, N-lim = nitrogen limitation, P-lim = phosphorus limitation, Redfield = Redfield ratio.

**
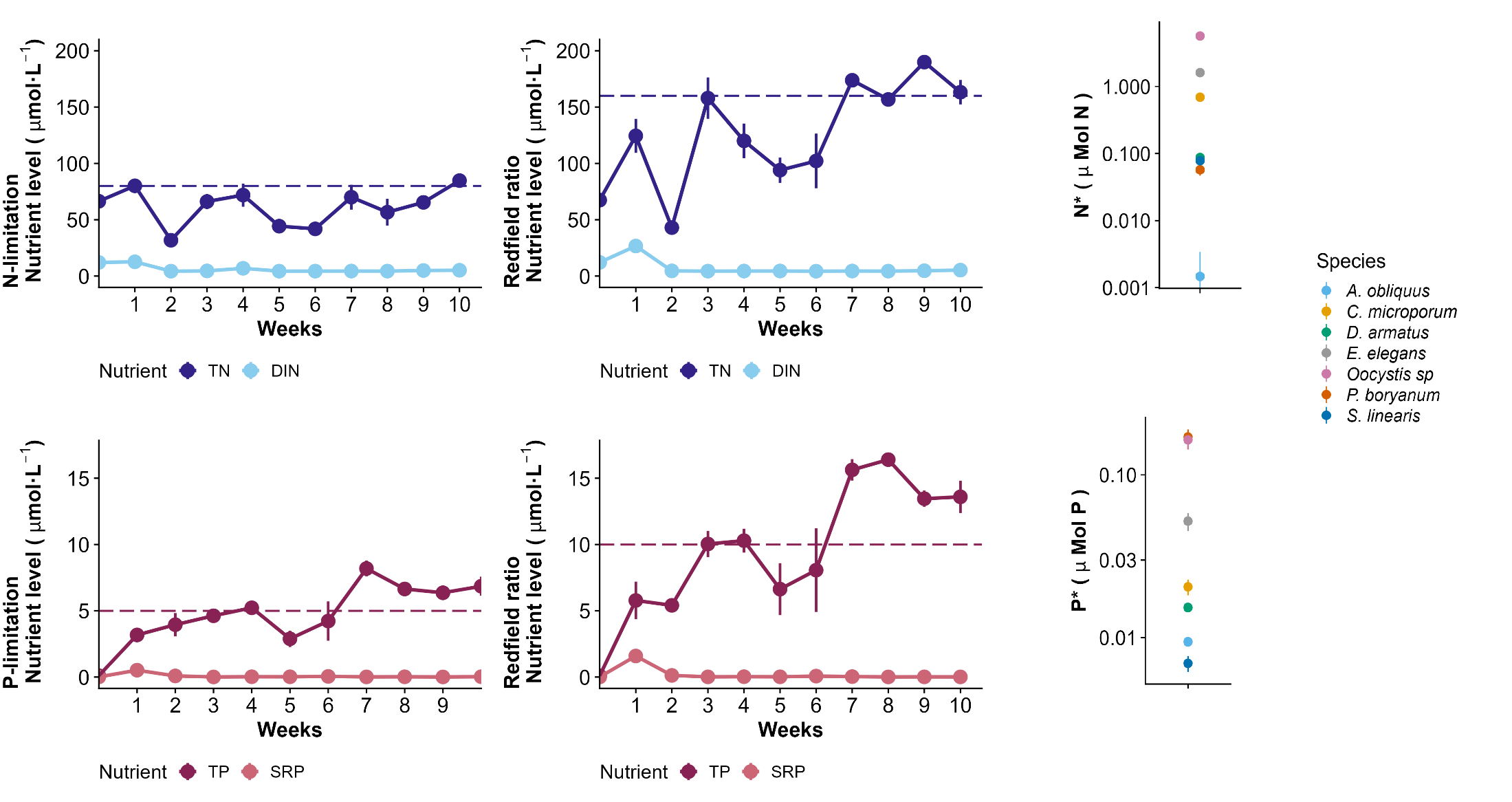
**

**SI 1A Figure 2.** Resource levels (total and dissolved nutrients) over time under resource-limitation treatments (left panels), and balance resource supply (Redfield ratio, central panels). Under balanced supply treatment, nutrient levels reached limiting values, suggesting co-limitation*. R** values calculated under these limiting conditions (right panels). All resource concentrations were calculated as the mean value for each mesocosms (n=4). Vertical bars represent standard error bars.

Abbreviations: TN = Total Nitrogen, DIN = Dissolved Inorganic Nitrogen, TP = Total Phosphorous, SRP = Soluble Reactive Phosphorous.

**B. Methodology of the laboratory experimental design**

Prior to each experiment, we inoculated all populations into liquid batch culture for acclimation to low-resource conditions, to prevent carryover effect from prior environments. All the strains were acclimated in 6-well plates to two different resource levels over 75 hours. The low resource acclimation conditions were set to match the lowest resource level in the *R** experiments to minimize transfer of nutrients from the acclimation culture to the experimental populations. The high resource acclimation level was set halfway between the lowest and highest resource level. The low resource acclimation levels for light, nitrogen and phosphorus were 8 μmol·m-2·s-1, 5 μM N and 0.5 μM P, respectively, where the high resource acclimation levels for light, nitrogen and phosphorus were 42 μmol·m-2·s-1, 80 μM N and 8 μM P. The acclimated strains were then diluted into well plates to achieve desired experimental resource levels. We used a handling robot TECAN EVO 100 to make dilutions, inoculations and randomization of the replicates into the plates.

For the determination of the minimum nitrogen requirements (*N**), the N levels were: 5, 10, 20, 40, 60, 80, 100, 400, 600 and 1000 μM N. For the determination of the minimum phosphorus requirements (*P**), the P levels were 0.5, 1, 2, 4, 6, 8, 10, 20, 35 and 50 μM P. For the calculation of the light requirements (*I**), N and P were 1000 μM N and 50 μM P respectively, and light was supplied at one of ten levels: 0.25, 1.5, 5, 12.5, 27.5, 50, 82.5, 125, 175 and 250 μmol photons/m2/s of PAR. We used neutral density filters (Solar Graphics Clearwater, USA), which alter the total amount of light supplied without changing light spectrum, to manipulate the light conditions in the light experiment. The experimental light intensities under the filters were measured with a Skye PAR Quantum sensor.

We grew our strains in 96-well plates that were incubated in temperature-controlled incubators (Multitron, Infors HT, Switzerland) at 20ºC and 100 rpm, with a full-day cycle and an irradiance of 150 µmol/m2/s (standard conditions), except for the light treatment. We inoculated the monocultures at a very low density (< 50 RFU) to track their growth until they reached the stationary state. We measured growth based on chlorophyll *a* relative fluorescence units (RFUs) over time, using a Biotek Cytation 5 plate-reader. Chlorophyll *a* fluorescence can be used as a proxy for algal biomass, especially when the growth is measured from low density until exponential phase. For each strain and resource level, four replicates of 30 RFUs each were transferred into 48 inner wells of 96-well plates, with a final volume of 125 µL per well. The outer wells of the plate were filled with 125 µL of sterilized water to prevent evaporative losses across the plate. Each plate was covered with a Breathe-Easy© gas-permeable membrane (Sigma-Aldrich).

Cell size was measured at the end of each *R** experiment (*I**, *N**, *P**). After the final RFU measurements, we fixed the populations in each well by adding a 10% glutaraldehyde fixative solution, and stored the plates at 4°C until later analysis. We estimated cell size at two different resource levels, *i.e.* full COMBO medium (150 µmol/m2/s, 1000 µM N, 50 µM P for the light, nitrogen and phosphorus experiment, respectively) and low resource conditions, *i.e.* the minimum resource level at which all species showed positive growth (10 µmol/m2/s, 20 µM N, 4 µM P for the light, nitrogen and phosphorus experiment, respectively). Images were taken with the Cytation 5 plate reader using bright field and fluorescence channel 1 (specific to capture chlorophyll *a*), using 20x magnification. Size values (biovolume) were automatically obtained for each individual present in the image with Gen5© software. Values were averaged for each well (strain replicate) within each strain and species.

Additionally, we performed a common garden experiment to determine the internal stoichiometric ratios C:N and C:P in all isolated strains. We grew them under two resource levels (the same levels as for image capturing). We inoculated 30 RFUs into tissue culture flasks with 150 mL of culture medium, after 75 hours of acclimation. Subsamples of 125 µL were taken daily to estimate growth curves. We allowed all the strains to grow until they reached their carrying capacity (two weeks after the initial inoculation approximately), but strains growing under balanced resource supply were harvested during the mid-exponential phase, whilst strains growing under low resource levels were harvested once the stationary phase was reached. Algal biomass was harvested by filtering each culture onto two ashed (400°C) Whatman® glass microfiber filters GF/F (one of grade 47 mm and one of grade 25 mm). We then dried the filters in an oven overnight at 60°C. The 47mm filter was used to estimate the elemental carbon and nitrogen content of the biomass on an Elementar vario PYRO cube EA-IRMS, and the 25 mm filter was used to estimate phosphorus content using Skalar San++Continuous Flow P/N analyzer. The phosphorus samples were first digested and completely oxidized using a peroxydisulfate solution.

**C. Monod curves for *I**, *N** and *P** for the seven studied species**

Colors show different treatments. L-lim = light limitation, N-lim = nitrogen limitation, P-lim = phosphorus limitation, Redfield = Redfield ratio.


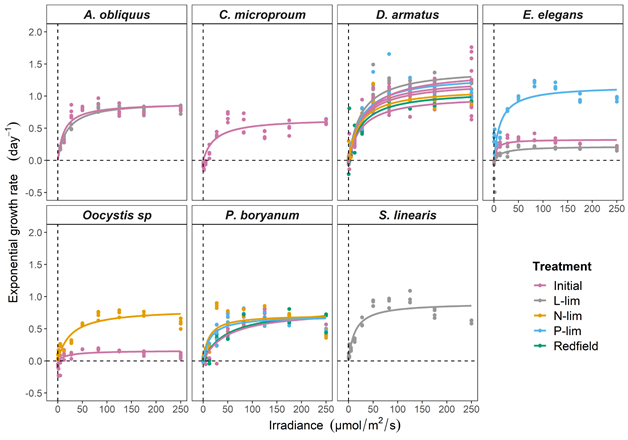


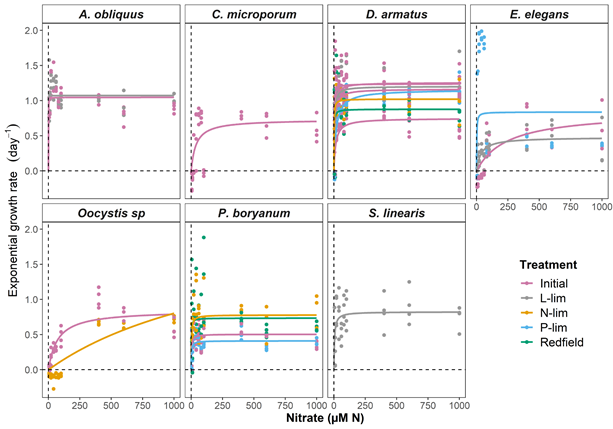


 
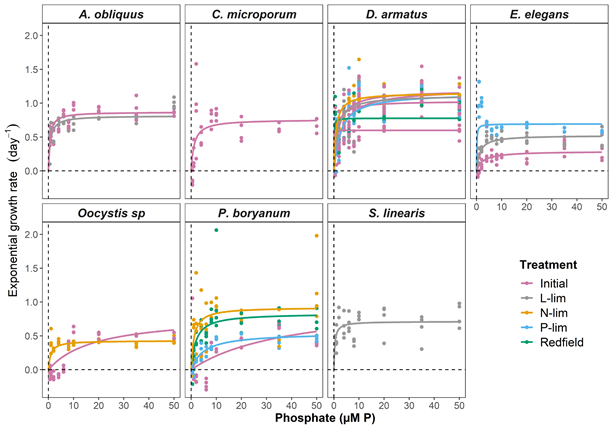

Supplement: Supplementary file 1 — Data S1 [file ELE-25-2397-s001.docx]
