# Supplementary material for: Ecology and evolution of competitive trait variation in natural phytoplankton communities under selection
Source: Ecol Lett. 2022 Sep 27;25(11):2397–409. doi: 10.1111/ele.14103 (PMC9828480; doi:10.1111/ele.14103)
Supplement: Supplementary file 2 — Data S2 [file ELE-25-2397-s002.docx]

**Supplementary Information 2**


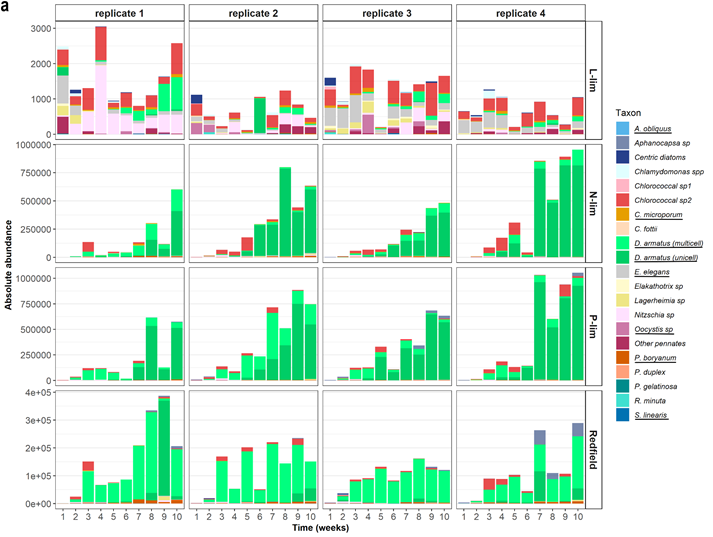


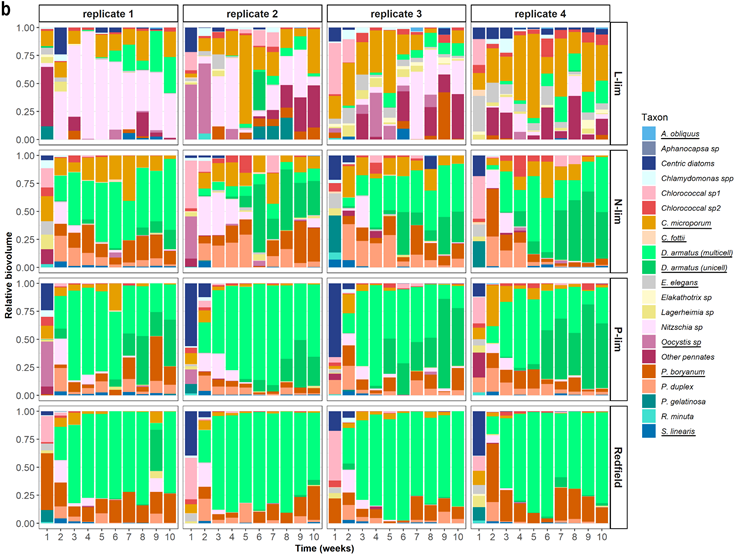


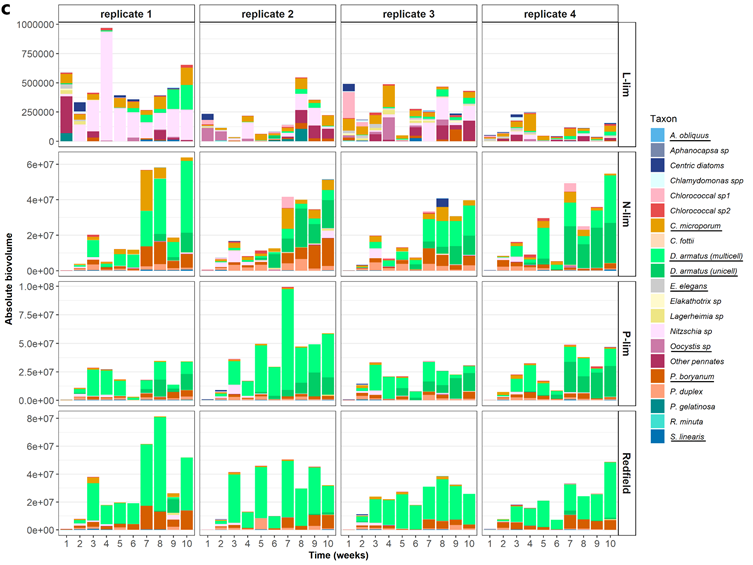


**SI 2 Figure 1.** Phytoplankton community composition over time. The x-axis represents time in weeks and the y-axis represents taxon absolute abundance (individuals·mL-1; panel a), relative biovolume and absolute biovolume (μm3·mL-1; panels b,c) at each treatment and replicate. Species that were isolated for laboratory experiments are underlined. The dominant taxon, *Desmodesmus armatus*, was also accounted for by its two different morphotypes (*Desmodesmus* unicellular, *Desmodesmus* multicellular, in green tonalities).


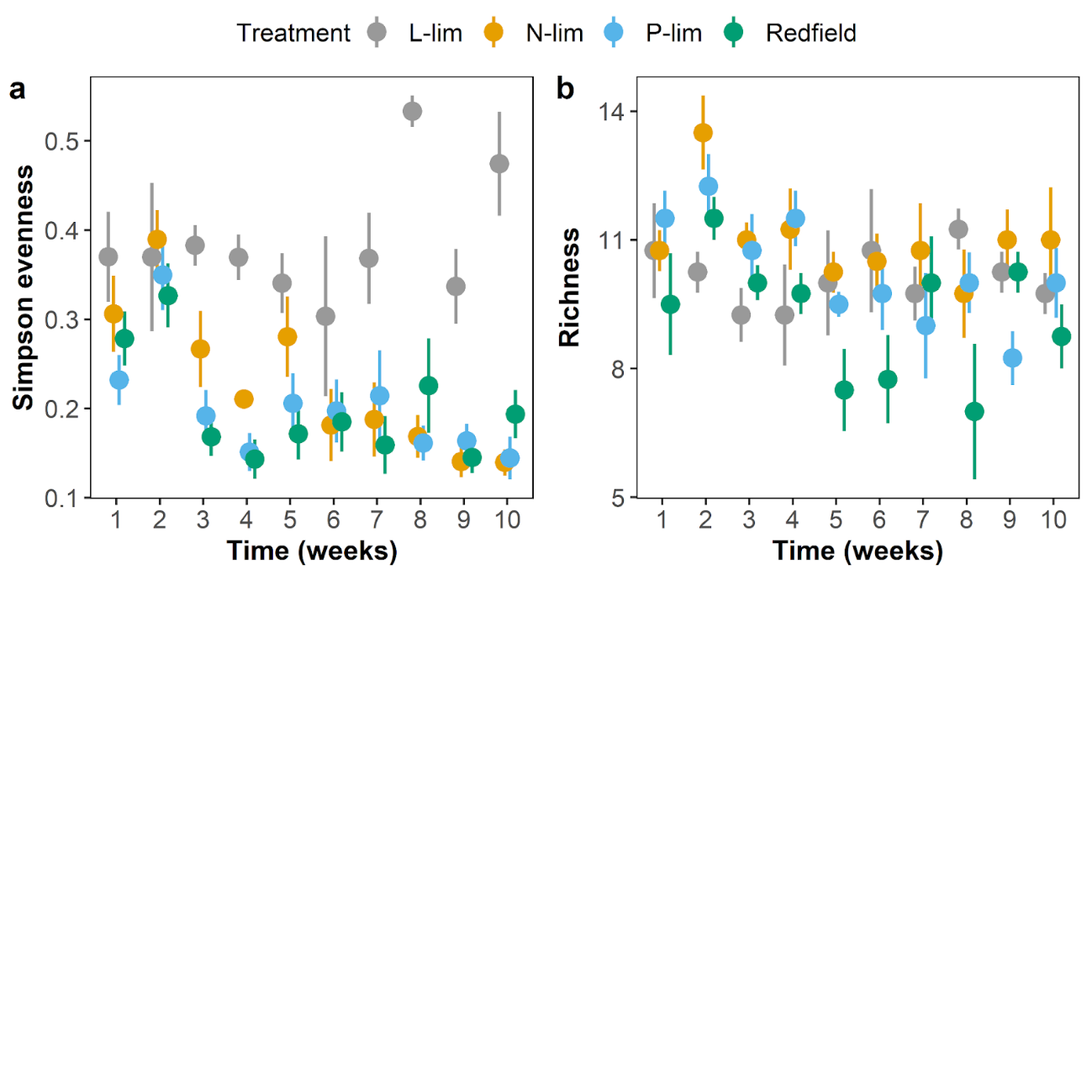


**SI 2 Figure 2.** Phytoplankton species evenness (Simpson’s index, a) and richness (b) over 10 weeks.

L-lim = light limitation, N-lim = nitrogen limitation, P-lim = phosphorus limitation, Redfield = Redfield ratio.


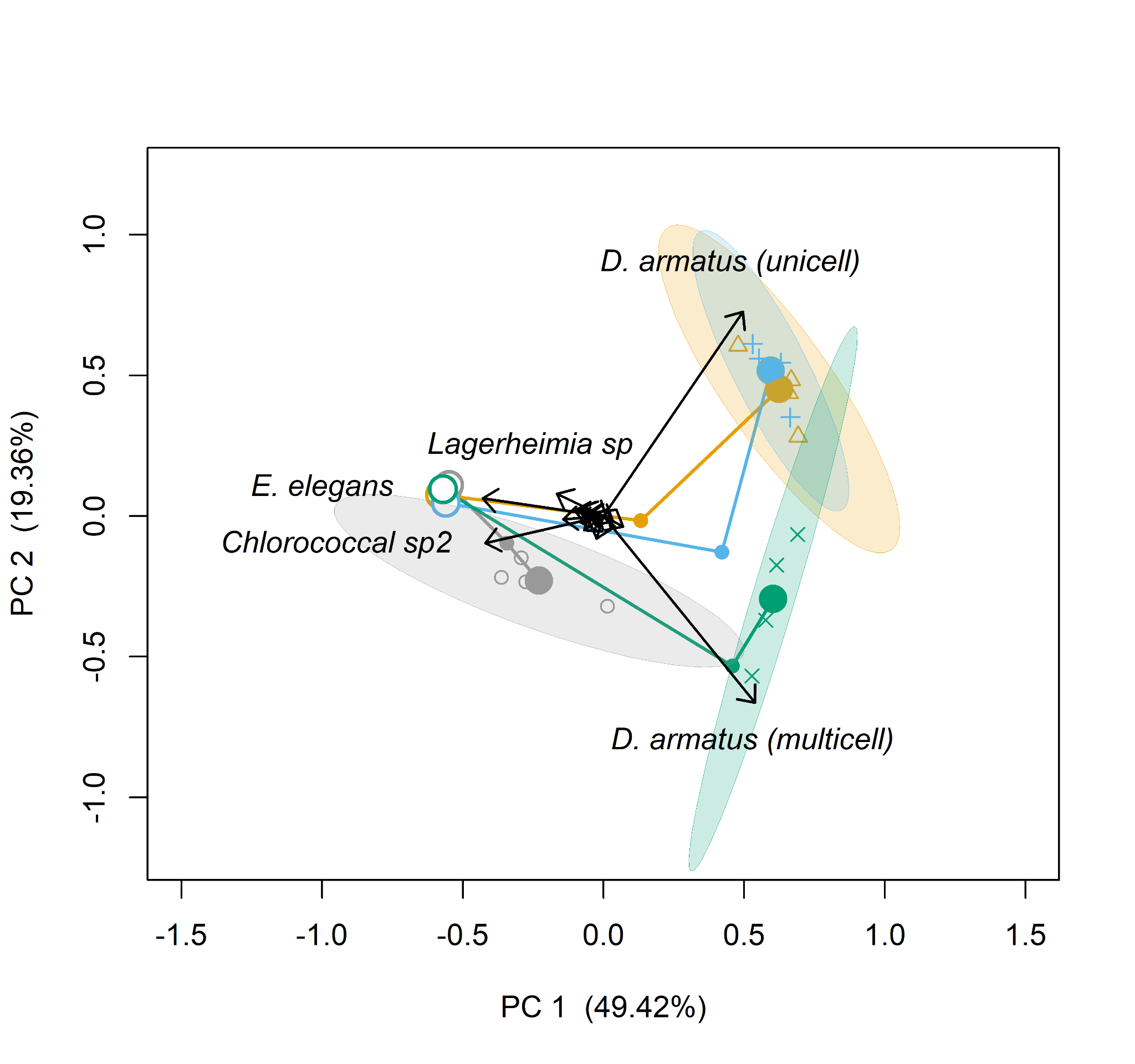


  
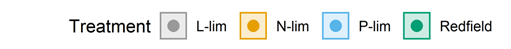


**SI 2 Figure 3.** Community Trajectory Analysis (CTA). Phytoplankton community abundance (individuals mL-1) of mesocosms showing differences in composition between light-limited and the other treatments. The length of the corresponding arrow indicates the weighting of the abundance of each species (Hellinger-transformed) on the principal component axes. The centroid of each treatment is shown for three separate time points: Open circles represent the starting time point (week 1), small closed circles indicate an intermediate time point (week 5) and large closed circles represent the final time point (week 10). Lines moving between the circles indicate the mean trajectory of community abundance composition. Ellipses indicate the confidence interval (95% CI) of the community composition for each treatment at the final time point, with four replicates for each treatment (each treatment replicate is represented with different signs and the same color of the treatment). L-lim = light limitation, N-lim = nitrogen limitation, P-lim = phosphorus limitation, Redfield = Redfield ratio.


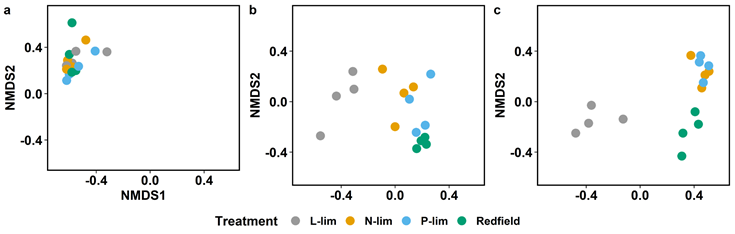


**SI 2 Figure 4.** Non-metric Multidimensional Scaling (NMDS) on phytoplankton species abundance. Representation of community composition’ ordinations of each mesocosm at the beginning of the experiment (week 1, panel a), during the experiment (week 5, panel b) and at the end of the experiment (week 10, panel c). Overall NMDS stress = 0.109.

L-lim = light limitation, N-lim = nitrogen limitation, P-lim = phosphorus limitation, Redfield = Redfield ratio.


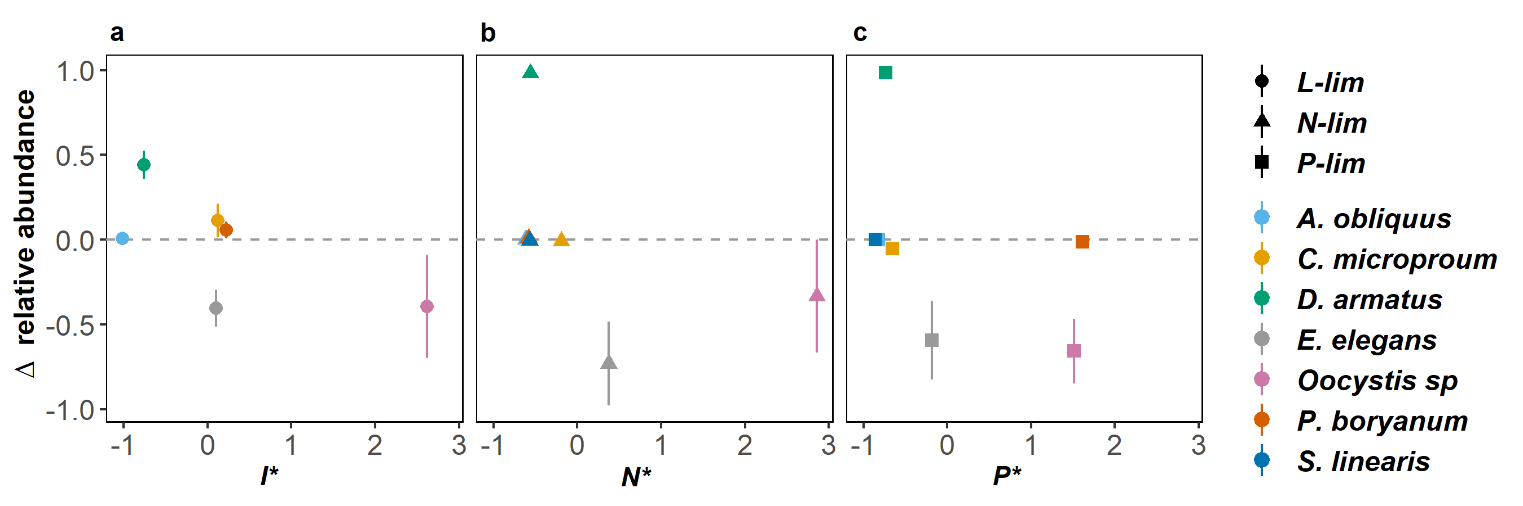


**SI 2 Figure 5.** Trait value versus change in species relative abundances over 10 weeks. *I** in panel a, *N** in panel b, and *P** in panel c. Error bars represent differences in relative abundances per replicate (n = 4) for each treatment. Species are represented by different colors.


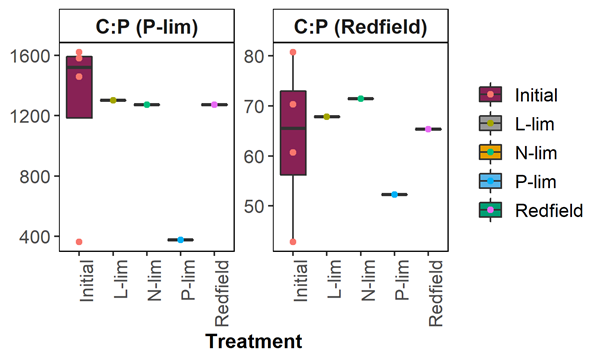


**SI 2 Figure 6.** Variation of C:P molar ratios in *D. armatus* strains across different treatments.  Left panel shows C:P ratio when strains were grown under P limitation. Right panel corresponds to C:P ratio when strains were grown under balanced resource supply (Redfield ratio).

| **Model** | **Variables** | **Covariate(s)** | **estimate** | ***p*-value** | **statistic** |
| --- | --- | --- | --- | --- | --- |
| **1** | ***P* - I**** | **-** | **0.4502** | **0.041**** | **2.1977** |
| **2** | ***P* - I**** | ***N**** | **0.3854** | **0.093 *** | **1.7721** |
| **3** | ***P* - I**** | ***µ_max_ max*** | **0.4177** | **0.067*** | **1.9507** |
| **4** | ***P* - I**** | **size** | **0.3999** | **0.08*** | **1.8515** |
| 5 | *P* - I** | *N*,* *µ_max_ max* | 0.35 | *n.s.* | 1.5403 |
| 6 | *P* - I** | *N*,* size | 0.3179 | *n.s.* | 1.3825 |
| 7 | *P* - I** | *µ_max_ max*, size | 0.3569 | *n.s.* | 1.5754 |
| 8 | *P* - I** | *N*,* *µ_max_ max*, size | 0.2689 | *n.s.* | 1.117 |
| **9** | ***N* - I **** | **-** | **0.3955** | **0.076*** | **1.8771** |
| 10 | *N* - I ** | *P** | 0.3149 | *n.s.* | 1.4077 |
| **11** | ***N* - I **** | ***µ_max_ max*** | **0.4019** | **0.079*** | **1.8624** |
| **12** | ***N* - I **** | **size** | **0.4082** | **0.074*** | **1.897** |
| 13 | *N* - I ** | *P*,* *µ_max_ max* | 0.3297 | *n.s.* | 1.434 |
| 14 | *N* - I ** | *P*,* size | 0.3289 | *n.s.* | 1.4358 |
| **15** | ***N* - I **** | ***µ_max_ max*, size** | **0.4169** | **0.076*** | **1.8914** |
| 16 | *N* - I ** | *P*,* *µ_max_ max*, size | 0.3489 | *n.s.* | 1.4892 |
| 17 | *N* - P** | - | 0.2787 | *n.s.* | 1.2649 |
| **18** | ***P** - size** | **-** | **0.3773** | **0.092*** | **1.7760** |
| **19** | ***P** - size** | ***N**** | **0.3937** | **0.086*** | **1.8174** |
| 20 | *P** - size | *I** | 0.3106 | *n.s.* | 1.3861 |
| **21** | ***P** - size** | ***µ_max_ max*** | **0.3838** | **0.095*** | **1.7636** |
| **22** | ***P** - size** | ***N*,* *µ_max_ max*** | **0.3997** | **0.09*** | **1.7981** |
| 23 | *P** - size | *I*,* *µ_max_ max* | 0.3141 | *n.s.* | 1.3642 |
| 24 | *P** - size | *N*, I** | 0.3285 | *n.s.* | 1.434 |
| 25 | *P** - size | *N*, I*,* *µ_max_ max* | 0.3344 | *n.s.* | 1.4191 |
| 26 | *I** - size | - | 0.2402 | *n.s.* | 1.0786 |
| 27 | *N** - size | - | -0.0029 | *n.s.* | -0.0127 |
| 28 | *P** - *µ_max_ max* | - | -0.1857 | *n.s.* | -0.8238 |
| **29** | ***I* - µ_max_ max*** | **-** | **-0.377** | **0.092*** | **-1.7741** |
| **30** | ***I* - µ_max_ max*** | ***N**** | **-0.3838** | **0.095*** | **-1.7635** |
| 31 | *I* - µ_max_ max* | *P** | -0.3344 | *n.s.* | -1.5052 |
| **32** | ***I* - µ_max_ max*** | **size** | **-0.3881** | **0.091*** | **-1.7867** |
| **33** | ***I* - µ_max_ max*** | ***N*,* size** | **-0.3975** | **0.092*** | **-1.7862** |
| 34 | *I* - µ_max_ max* | *P*,* size | -0.3430 | *n.s.* | -1.5058 |
| 35 | *I* - µ_max_ max* | *N*, P** | -0.3481 | *n.s.* | -1.5312 |
| 36 | *I* - µ_max_ max* | *N*, P*,* size | -0.3621 | *n.s.* | -1.5538 |
| 37 | *N*- µ_max_ max* | - | -0.0636 | *n.s.* | -0.2778 |
| 38 | *µ_max_ max* -size | - | -0.0009 | *n.s.* | -0.0039 |

**SI 2 Table 1.** Summary of estimates, *p*-value and statistics of correlation models between traits and partial correlations models with one, two or three covariates. In all cases, the correlation method was Pearson. Prior to analyses, variables were log_10_ transformed. Models with significant correlations are in bold.

Significance levels: ** *p*-value < 0.05, * *p*-value < 0.1, *n.s.* not significant.

| Model | Predictor | Intercept | Estimate | R2 | Adj R2 | *p*-value | AICc |
| --- | --- | --- | --- | --- | --- | --- | --- |
| 1 | *µ_max_ max* | - 1.8813 | 2.022 | 0.7666 | 0.689 | 0.052***** | 31.83 |
| 2 | *P** | 0.0844 | -1.187 | 0.0225 | -0.222 | *n.s.* | 27.69 |
| 3 | *I** | 0.2073 | -0.413 | 0.0428 | -0.196 | *n.s.* | 27.56 |
| 4 | *N** | 0.0809 | -0.06 | 0.0475 | -0.191 | *n.s.* | 27.54 |

**SI 2 Table 2.** Summary of regression models (linear modelling) to estimate the best trait predicting the variation of relative abundance in our mesocosm experiments. Predictor variables and their estimates, R^2^, Adjusted R^2^, *p*-value and Akaike Information Criterion corrected for small sample size (AICc) are depicted. Only species’ maximum growth rate (*µ_max_ max)* (model 1) was able to predict Δ relative abundance. Prior to analyses, variables were log_10_ transformed.

Significance levels: ** *p*-value < 0.05, * *p*-value < 0.1, *n.s.* not significant.
